# Mechanisms of Nerve Damage in Neuropathies Associated with Hematological Diseases: Lesson from Nerve Biopsies

**DOI:** 10.3390/brainsci11020132

**Published:** 2021-01-20

**Authors:** Chiara Briani, Sergio Ferrari, Marta Campagnolo, Matteo Tagliapietra, Francesca Castellani, Alessandro Salvalaggio, Sara Mariotto, Andrea Visentin, Tiziana Cavallaro

**Affiliations:** 1 Department of Neurosciences, University of Padova, 35100 Padova, Italy; marta.campagnolo@unipd.it (M.C.); francescacastellani2@gmail.com (F.C.); salvalaggio.a@gmail.com (A.S.); 2 Neurology Unit, Department of Neuroscience, Biomedicine and Movement Sciences, University of Verona, 37134 Verona, Italy; sergio.ferrari@aovr.veneto.it (S.F.); matteo.tagliapietra@univr.it (M.T.); sara.mariotto@gmail.com (S.M.); tiziana.cavallaro@aovr.veneto.it (T.C.); 3 Hematology and Clinical Immunology Unit, Department of Medicine, University of Padova, 35100 Padova, Italy; andrea.visentin@aopd.veneto.it

**Keywords:** peripheral neuropathy, sural nerve biopsy, neurolymphomatosis, lymphoma, monoclonal gammopathy

## Abstract

Despite the introduction of non-invasive techniques in the study of peripheral neuropathies, sural nerve biopsy remains the gold standard for the diagnosis of several neuropathies, including vasculitic neuropathy and neurolymphomatosis. Besides its diagnostic role, sural nerve biopsy has helped to shed light on the pathogenic mechanisms of different neuropathies. In the present review, we discuss how pathological findings helped understand the mechanisms of polyneuropathies complicating hematological diseases.

## 1. Introduction

Sural nerve biopsy has long been a valuable diagnostic tool for the study of peripheral neuropathies [1], although the recent introduction of non-invasive techniques (e.g., neuroimaging techniques, skin biopsy) [2] and advanced molecular testing has changed the diagnostic workup of peripheral nervous system diseases.

Recently, it has been shown that serum neurofilament light chain (NfL), a biomarker of axonal nerve damage, correlates with axonal loss from sural nerve biopsies, thus providing pathological evidence of the validity of NfL in quantifying axonal damage in peripheral neuropathies [3].

In hematological diseases, sural nerve biopsy remains the gold standard for the diagnosis of vasculitis, neurolymphomatosis, and light chain amyloidosis. Whole sural nerve biopsy is required to assess these pathologies frequently characterized by epineurial lesions and are processed according to routine procedures [4]. Besides its diagnostic role, sural nerve biopsy has helped understand the pathogenesis of several neuropathies, first of all, the most common immune-mediated neuropathy, that is the neuropathy with antibodies to the myelin-associated glycoprotein (anti-MAG neuropathy).

In the present review, we discuss what we have learned from nerve biopsies on the mechanisms of nerve damage in neuropathies associated with hematological diseases. The results are described in detail in Supplementary Materials Table S1.

## 2. Polyneuropathy with Antibody to Myelin-Associated Glycoprotein (MAG)

Anti-myelin-associated glycoprotein (anti-MAG) neuropathy is a chronic demyelinating sensorimotor polyneuropathy associated with IgM monoclonal gammopathy and is the most common paraproteinemic neuropathy. In anti-MAG antibody neuropathy, the paraprotein is associated with monoclonal gammopathy of undetermined significance (MGUS) in approximately 80% of patients, and with Waldenström macroglobulinemia (WM) in the remaining 20% [5]. Slowly progressive sensory ataxia, upper limb tremor, and decrease in vibration perception are the characteristic clinical symptoms, with findings of a large fiber, symmetric sensorimotor neuropathy with markedly increased distal latencies on nerve conductions studies. Clinically, however, motor involvement occurs only late in the course of the disease, if ever present [6].

The neuropathy was first described by Latov in 1980 as a neuropathy caused by an IgM monoclonal peak reacting against peripheral nerve myelin [7], with MAG later identified as the molecular target [8]. MAG is a 100 kDa glycoprotein, made of five extracellular Ig-like domains, a transmembrane and a cytoplasmic domain, localized on the membranes of Schwann cells and oligodendrocytes [9]. The protein mediates axoglial interactions through the binding to an axolemma receptor, conditioning the myelination maintenance and tropism of the axon. Indeed, MAG-deficient mice, although showing a normal myelination process, later develop a peripheral neuropathy with myelin degeneration and neural cytoskeleton alteration with consequent reduction in axon caliber [10,11]. The IgM paraprotein attacks MAG in a glucidic epitope, namely the carbohydrate CD57/HNK-1, as purified human MAG no longer shows anti-MAG reactivity after deglycosylation [12]. Sulfoglucuronyl paragloboside (SGPG), a glycolipid found only in peripheral nerves and sharing the CD57/HNK-1 epitope observed in MAG, is a possible alternative target [13,14]. The binding of IgM antibodies to MAG/SGPG causes a complement-mediated widening of external myelin lamellae, and consequent nerve demyelination [15]. Pathological studies of sural nerves show mild loss of myelinated fibers, segmental demyelination, and occasional focal intramyelin edema on teased nerve fibers, with deposits of IgM and complement in myelin sheets on immunofluorescence studies. Intraperiodic line splitting and widening of myelin lamellae are reported in ultrastructural studies [16,17]. Endoneurial immunoglobulin deposits were observed not only in anti-MAG antibody neuropathy but also in some cases of neuropathy with MGUS or hematologic malignancies and it is likely that the incidence of peripheral neuropathy is associated with endoneurial immunoglobulin deposits is underestimated [18].

There is strong evidence supporting the pathogenetic role of anti-MAG/SGPG antibodies:

- pathological studies of sural nerves show deposits of IgM and complement in myelin sheets, suggesting the need for complement activation in the demyelination process [19];
- IgM recognize NCAM (Neural Cell Adhesion Molecules) and are seen in correspondence of MAG in demyelinated areas [13]; in skin biopsies of the same patients there is a concurrent localization of IgM, C3d complement, and MAG in the dermal myelinated fibers, leading to the loss of nerve fibers [20];
- feline nerves injected with the serum of patients with anti-MAG/SGPG IgM supplemented with additional complement, develop complement-mediated demyelination and conduction block within 2–9 days [21];
- systemic transfusion of chickens with anti-MAG IgM produces segmental demyelination with IgM deposits on external myelin sheets and consequent widening of myelin lamellae as observed in human pathology [22];
- cats immunized with purified SGPG develop an ataxic neuropathy with the involvement of dorsal root ganglia, similar to anti-MAG antibody neuropathy [23];
- patients with anti-MAG antibody neuropathy respond to immunomodulant therapies, especially monoclonal antibodies (i.e., rituximab, obinutuzumab, ibrutinib) [24,25,26], and therapy response seems to correlate with the reduction of anti-MAG antibodies titers [27,28,29].

**Figure 1:**
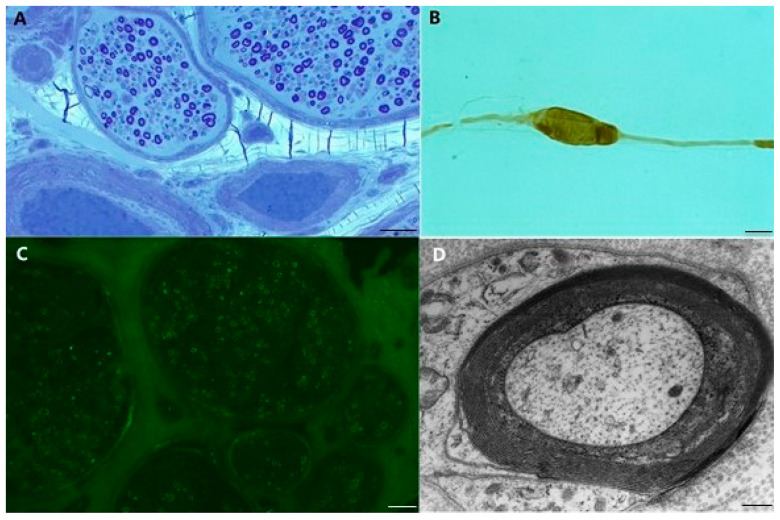
Sural nerve biopsy from anti-MAG antibody neuropathy. (**A**) Slight reduction of myelinated fibers is observed; rare fibers are surrounded by disproportionately thin myelin sheaths compared to axonal diameter and a few fibers show axonal degeneration. A myelinated fiber with intramyelinic oedema is evident. (semithin section, toluidine blue; original magnification 20×; bar 50 μm); (**B**) Teased fiber showing a focal pale swelling in the remyelinating internode (original magnification 40×; bar 25 μm); (**C**) Direct Immunofluorescent studies on frozen sections shows the presence of IgM deposition on the myelin sheaths of several fibers (original magnification 20×; bar 50 μm); (**D**) Electron micrograph of a transverse section through a nerve fiber with widening of myelin lamellae as a result of separation along intraperiod line. (original magnification 12,000×; bar 1 μm).

Pathological findings from sural nerve biopsies of anti-MAG antibody neuropathy are represented in Figure 1.

## 3. Cryoglobulinemic Neuropathies

A rare cause of axonal neuropathy is cryoglobulinemia, which is an inflammation of small blood vessels caused by peculiar immunocomplexes called cryoglobulins. Cryoglobulins usually clump together below 37 °C, activate the complement cascade, and recruit acute phase blood cells inflating and damaging capillaries of the extremities but also of the skin, the glomerulum and vasa nervorum. In the context of hematological diseases, both type I (monoclonal IgMs or IgGs, rarely IgA) and type II cryoglobulinemia (mixed forms, usually associated with HCV infections or connective tissue diseases occur in MGUS, or B cells malignancies (WM, chronic lymphocytic leukemia, CLL), with a predominance of type II cryoglobulinemia in WM [29,30]. Type III cryoglobulinemia caused by polyclonal IgM and IgG is seen predominantly in chronic infections (mainly hepatitis C virus, HCV) and also connective tissue diseases (in particular Sjogren syndrome) [31,32,33]. In both Type II and III the IgM antibodies expressing activity against the Fc portion of IgG are called rheumatoid factor. Monoclonal IgMs cryoglobulins cause a severe painful neuropathy, that may present with multifocal distribution also involving cranial nerves. Cryoglobulinemia may be associated with arthralgia, glomerulonephritis, and dermatological findings such as skin ulceration or purpura. On sural nerve biopsy, perivascular lymphomonocytic infiltration is associated with tunaca media necrosis, characteristic for vasculitis, petechiae, focal loss of myelinated and unmyelinated fibers often with active axonal degeneration or regeneration depending on the stage of the disease.

Cryoglobulin-associated neuropathy also benefits from rituximab, as well as from steroids and plasma-exchange in case of life-threatening manifestations [31].

**Figure 2:**
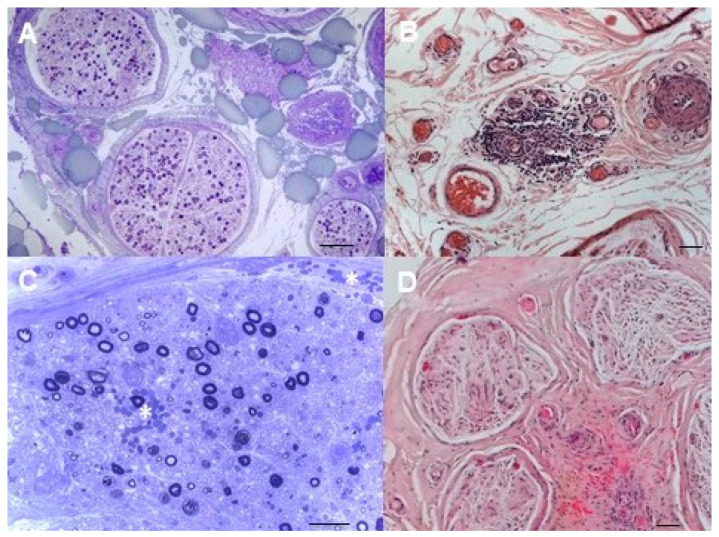
Sural nerve biopsy from cryoglobulinemic neuropathy. (**A**) Asymmetric focal loss of myelin fibers and epineurial perivascular inflammatory infiltration are evident. (semithin section, toluidine blue, original magnification, 10×; bar 100 μm). (**B**) A perivascular infiltrate of lympho-monocytes is recognizable in epineurium. (paraffin section, H&E, original magnification 20×; bar 50 μm). (**C**) Active axonal degeneration characterizes the pathological process together with endoneurial and subperineurial petechiae (*) (semithin section, toluidine blue, original magnification 40×; bar 50 μm). (**D**) Haemorrhagic suffusions (petechiae) are also present in epineurium (paraffin section, H&E, original magnification 10×; bar 50 μm).

Pathological findings from sural nerve biopsies of patients with cryoglobulinemic neuropathy are represented in Figure 2.

## 4. Neurolymphomatosis

The term “peripheral lymphomatosis” was firstly proposed by Lhermitte and Trelles to describe a 67-year-old woman with malignant neuropathy and lymphoid nerve infiltrates but no systemic or brain involvement [30,34,35]. Neurolymphomatosis is, therefore, a pathological definition and represents a rare neurologic manifestation of hematological malignancies in which cranial or peripheral nerve roots, plexi, or nerves are infiltrated by lymphomatous or leukemic cells. Rarely, neurolymphomatosis is the primary manifestation of non-Hodgkin lymphomas (NHL), while more commonly, neoplastic cells disseminate into the peripheral nervous system from systemic sites or central nervous system [30]. Neurolymphomatosis may be also the first manifestation of the relapse of the underlying lymphoma. Diffuse large B-cell lymphoma (DLBCL) is the leading cause of neurolymphomatosis, but any lymphoma may be because of neurolymphomatosis.

In a case series by Grisariu et al., neurolymphomatosis was associated with NHL in 90% of 50 patients [36], with the remaining patients presenting with an underlying B-cell acute leukemia. Among NHL, almost 3 out of 4 cases were DLBCL, 9% were follicular lymphoma whereas peripheral T-cell lymphoma and mantle cell lymphoma were observed rarely. Neurolymphomatosis was primary in almost one-third of cases in the same series, while another study by Baehring et al. reported more than 80% of patients without prior history of lymphoma [34]. Primary neurolymphomatosis at onset in CLL and, rarely, in natural killer cell lymphoma [37,38,39,40] has also been reported. The rarity of the disease as well as the heterogeneity of the clinical presentation (depending on the location of the malignant cells in the peripheral nervous system) together with the technical difficulties to establish a definitive diagnosis justify the delay between onset of symptoms and diagnosis, which requires nerve biopsy. In a study by Tomita et al. [41], the most frequent misdiagnosis was chronic inflammatory demyelinating polyradiculoneuropathy (CIDP) due to the presence of demyelinating features [42]. Neurolymphomatosis should therefore be considered in any patient with unexplained peripheral nervous system manifestations. Neurophysiology is needed to define the type of nerve damage and to identify the best candidate nerve for biopsy. Although magnetic resonance imaging (MRI), Positron emission tomography with computed tomography (PET-CT) and PET-MRI neuroimaging advances [37,43,44] the histological examination of the nerve remains the gold standard for the diagnosis, showing malignant lymphocytes infiltrate which may be located in the perineurium, epineurium, and endoneurium [45]. In our experience, asymmetric axonal loss is secondary to a focal monomorphic lymphocytic infiltration, predominantly epineurial, that is monoclonal on immunohistochemical cell typing [30,35,37]. Flow cytometry on cerebrospinal fluid also emerged as a possible diagnostic investigation, useful for the characterization of the malignant clonal lymphocytes [30,45]. Detection of a clonal B or T-cell receptor gene may help confirm lymphomatous infiltration in the nerve biopsy. This is commonly performed by standard PCR techniques designed to assess the diversity of the junctional regions in Ig or T-cell receptor genes [46].

**Figure 3:**
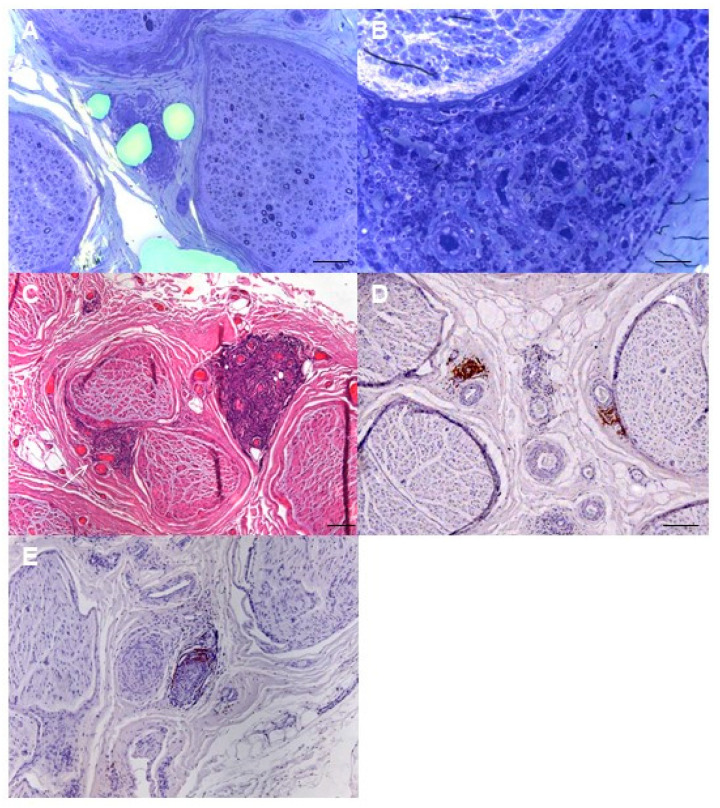
Sural nerve biopsy from patients with neurolymphomatosis. (**A**) Epineurial monomorphic perivascular infiltrate of lympho-monocytes and mild loss of myelin fibers in nerve fascicles are observed (semithin section, toluidine blue, original magnification 20×; bar 100 μm). (**B**) Diffuse lymphomatous infiltration involves the epineurium and perineurium (semithin section, toluidine blue, original magnification 40×; bar 50 μm). (**C**) The epineurium is infiltrated by a voluminous monomorphic lymphoid cell accumulation; minor infiltrates are also observed in perivascular sites (arrows) (paraffin section, H&E, original magnification 10×; bar 100 μm). (**D**,**E**) Immunocytochemistry with anti-CD20 antibodies demonstrates a prevalence of B lymphocytes in epineurial infiltrates in two different cases of neurolymphomatosis (paraffin sections, original magnification 20×; bar 100 μm).

Pathological findings from sural nerve biopsies of patients with neurolymphomatosis are represented in Figure 3.

## 5. Amyloidosis

Nerve biopsy represents the gold standard to demonstrate amyloid deposits in patients with amyloid neuropathy, both in immunoglobulin light-chain (AL) amyloidosis and transthyretin (TTR)-related amyloid neuropathy [47]. After the demonstration of amyloid deposits by Congo red staining, amyloid typing with immunocytochemistry and identification of amyloid fibrils on electron microscopy should be used to formulate the correct diagnosis and start proper therapy. Sometimes, when the coexistence of the two conditions cannot be excluded a priori electron microscopy or immunohistochemistry are mandatory to identify the fibrils’ composition [47,48].

Asymmetric loss of nerve fibers parallels clinical symptoms, being extreme in advanced cases, and tend to affect unmyelinated and small myelinated fibers earlier [49]. Active axonal degeneration is often observed, together with concomitant signs of axonal regeneration. Segmental demyelination is sometimes observed on teased fibers in TTR-related amyloid neuropathy and leads to the speculation of a Schwann cell involvement in the pathogenesis of amyloid neuropathy. Amyloid deposits are typically stained by Congo red and show the characteristic apple-green birefringence under polarized light. In early forms, the deposition is localized mainly in small vessel walls but extends to subperineural and epineural regions in advanced cases [50]. Immunohistochemistry with antibodies against lambda or kappa chains, transthyretin, or other amyloidogenic proteins could then be used to characterize the disease.

The neuropathological findings in a large cohort of transthyretin (TTR)-related amyloid neuropathy have been recently reported [51]. Data from 60 sural nerve biopsies showed evidence of axonal loss, which was severe in the majority of cases (70%). Congo red staining was positive in 72.5% of the patients, mostly in Val30Met cases. When performed, immunohistochemistry with anti-TTR was positive in all patients, with four cases also cross-reacting with anti-light chains (in two of them, an MGUS was found) [45].

Nerve biopsy also helped shed light on the pathophysiology of TTR amyloidosis. The finding of C-terminal fragments TTR besides full-length TTR in ATTRv specimens led to differentiate two types of amyloid with different phenotype and likely therapy response [51,52]. Moreover, data from experimental models helped understand the physiological role of TTR in neuroprotection [53].

**Figure 4:**
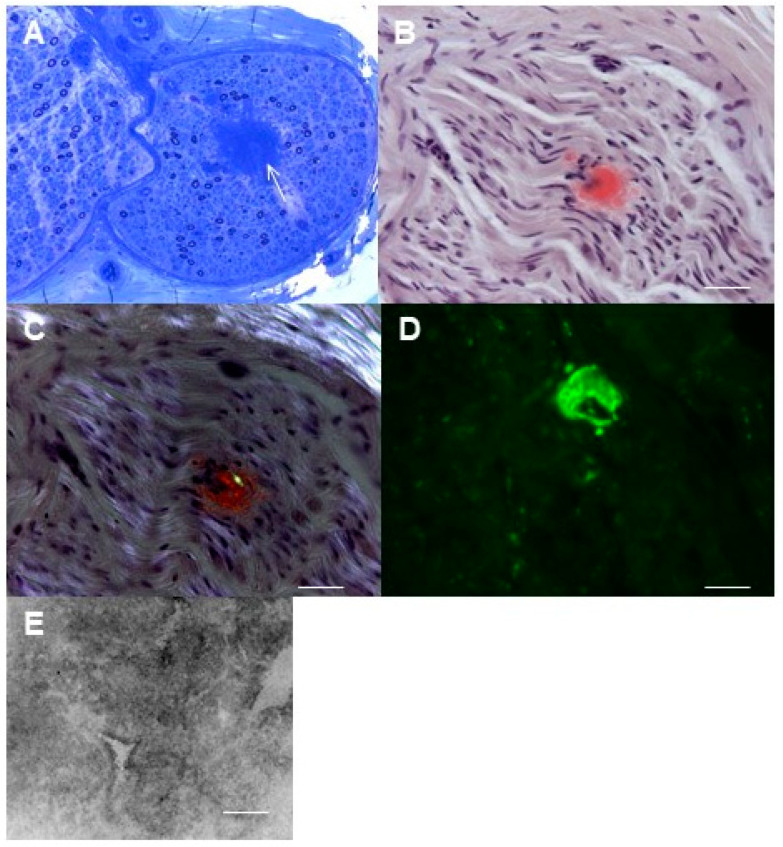
Sural nerve biopsy from patients with light chain amyloidosis (AL). (**A**) Axonal neuropathy with loss of myelinated nerve fibers and endoneurial perivascular accumulation of amorphous material (arrow) is observed (semithin section, toluidine blue, original magnification 20×; bar 100 mm). (**B**) Congo-red staining shows amyloid deposits in nerve fascicles (**B**), green birefringency is characteristically observed on cross polarizing filters (**C**) (paraffin section, original magnification 40×; bar 50 mm). (**D**) Direct immunofluorescence on frozen sections shows perivascular deposits of light chains (original magnification 40×; bar 50 μm). (**E**) Electron micrograph of endoneurial amyloid deposit composed of a dense aggregate of straight fibrils (original magnification 7000×, bar 1 μm).

Pathological findings from sural nerve biopsies of patients with AL amyloidosis are represented in Figure 4.

## 6. POEMS Syndrome

POEMS (Polyneuropathy, Organomegaly, Endocrinopathy, Monoclonal gammopathy, Skin changes) syndrome is a rare disorder associated with plasma cell dyscrasia. The hematological disorder (IgG or IgA paraprotein, lambda-restricted, with elevated serum free light chains but conserved κ/λ ratio), together with the polyneuropathy constitutes the mandatory criteria for diagnosis [54]. High serum or plasma levels of Vascular Endothelial Growth Factor (VEGF), which acts as a modulator of angiogenesis and vascular permeability, are detected in POEMS patients [55,56]. Moreover, VEGF is a reliable biomarker that can contribute to differentiate POEMS syndrome from other conditions with plasma cell dyscrasias and peripheral neuropathy, namely immunoglobulin AL amyloidosis or multiple myeloma [57].

One of the clinical hallmarks of the disease is the subacute sensory-motor polyneuropathy with prominent neuropathic pain, that can rapidly progress towards a significant motor impairment [58,59,60]. Neurophysiology commonly discloses mixed axonal and demyelinating pattern, that can favor the misdiagnosis with CIDP, despite the availability of reliable diagnostic criteria [61,62]. In line with this, nerve biopsies reveal prominent axonal degeneration as the main feature, with myelinated fiber loss and increased epineurial blood vessels [60,63], together with uncompacted myelin lamellae indicating demyelination. Moreover, abnormal findings in the blood–nerve barrier including the proliferation of endothelial cells, increased basal lamina thickness, and narrowed endoneurial vessels, were associated with higher values of serum VEGF and with increased VEGF expression in blood vessels, supporting its major role in the development of neuropathy [29,56]. Pachymeningeal non-inflammatory involvement has been reported in POEMS patients undergoing brain magnetic resonance. Histopathological studies disclosed hyperplasia of meningothelial cells, obstructive vessel disease due to endothelial proliferation, and signs of neovascularization, all features supporting a possible role of VEGF in the context of meningeal involvement [64,65].

**Figure 5:**
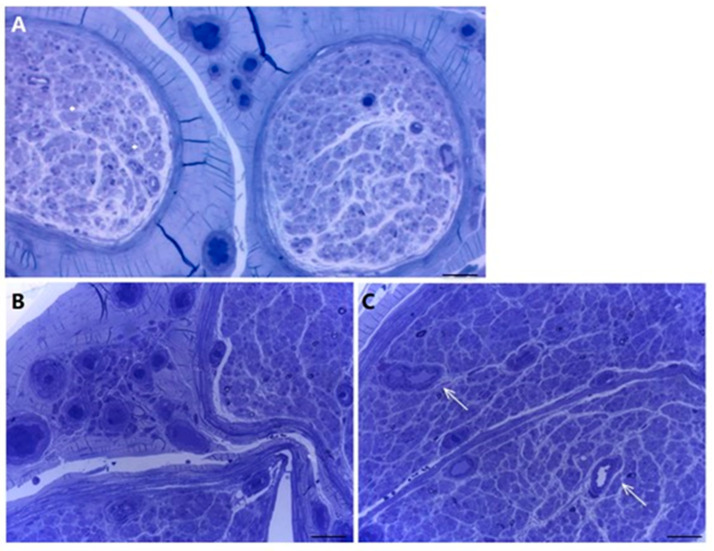
Sural nerve biopsy from a patient with POEMS syndrome. (**A**) Severe loss of myelinated fibers and occasional early remyelinating fibers (*) are observed (semithin section, toluidine blue, original magnification 20×; bar 100 μm). (**B**) Epineurial capillaries are surrounded by slight lymphomonocytic infiltrates (semithin section, toluidine blue, original magnification 40×; bar 100 μm). (**C**) Endothelial proliferation of endoneurial vessels is evident (arrows) (semithin section, toluidine blue, original magnification 40×; bar 100 μm).

Pathological findings from sural nerve biopsies of POEMS syndrome are represented in Figure 5.

## 7. Chemotherapy-Induced Neurotoxicity (CIPN)

Chemotherapy-induced peripheral nerve involvement, a dose-limiting effect possibly leading to treatment discontinuation, is common in the context of hematological disorders with an overall incidence of 60%. Clinical and neurophysiological examination disclose predominant sensory damage, presenting either as a length-dependent distal axonal neuropathy (involving large myelinated Aß fibers) or as a ganglionopathy with prominent dorsal root ganglia dysfunction (likely due to their anatomical structure with a less developed blood-nerve barrier) [30,66].

Several potentially neurotoxic drugs, such as vinca alkaloids, thalidomide, lenalidomide, proteasome inhibitors, and brentuximab vedotin are included in chemotherapy regimens for the treatment of different hematological conditions. Moreover, a new class of compounds, namely the immune checkpoint inhibitors monoclonal antibodies nivolumab and pembrolizumab, approved for the treatment of relapsed/refractory Hodgkin’s lymphomas, have been reported to potentially trigger an immune-mediated reaction involving the peripheral nervous system [30,66,67,68].

Sural nerve biopsies can provide information on possible pathogenic mechanisms of CIPN [30].

Vinka alkaloids (vincristine, vinblastine, vinorelbine, vindesine) are widely used in several chemotherapy protocols both in the adult and pediatric population. Through their ability to bind tubulin, they block microtubule formation and induce cell apoptosis, with subsequent impairment of axonal transport and secondary large fiber degeneration, resulting in a length-dependent axonal sensory-motor peripheral neuropathy. Cranial nerve neuropathies have also been reported, together with small fiber dysfunction with neuropathic pain and autonomic symptoms [69,70,71,72]. In addition to the main mechanism of damage, peripheral nerve involvement may be sustained also by mitochondrial dysfunction and abnormal calcium homeostasis [73]. A limited number of pathological studies were performed in animal models [74,75,76] confirming that vinka alkaloids determine severe axonal degeneration in peripheral nerves.

Thalidomide, a glutamic acid derivative, is employed in hematological conditions including multiple myeloma for its anti-angiogenic, immunomodulatory, and anti-inflammatory properties [77]. The mechanisms of thalidomide-induced neuropathy are still unclear, but different hypothesis have been suggested, including microvascular damage due to the anti-angiogenic properties of the drug, direct toxic effect [78], or inhibition of NF-kB, an essential regulator in the nerve growth factor-mediated effects on the survival of sensory neurons [79]. Histopathological studies on sural nerve biopsies from patients with thalidomide-induced neuropathy disclose heterogeneous findings, including selective loss of large fibers without segmental demyelination or inflammation, and signs of regeneration [80,81].

Lenalidomide, a thalidomide analog with immune-modulating and anti-angiogenetic activities employed in patients with multiple myeloma, has shown a more satisfying safety profile, especially regarding neurotoxicity when compared with its analog or with other drugs such as bortezomib [82]. In support of this, lenalidomide has also successfully been used in patients with POEMS syndrome [83], where neurotoxicity should be avoided since it can worsen the underlying peripheral neuropathy, which is often the main symptom in this condition.

Proteasome inhibitors bortezomib and carfilzomib are currently included in the treatment protocols for multiple myeloma, WM, and mantle cell lymphoma. Moreover, bortezomib-based regimens are an effective and safe treatment option also in patients with POEMS syndrome [77,84]. Bortezomib bears a severe neurotoxicity potential, with the vast majority of patients presenting with an axonal sensory-motor peripheral neuropathy and prominent neuropathic pain [85], and only a minority showing a predominant motor involvement and demyelinating features at neurophysiology and nerve biopsy [86]. Peripheral neuropathy has been suggested to develop due to bortezomib’s interference on transcriptional programs in neurons of the dorsal root ganglia with subsequent activation of neuroinflammatory pathways and secondary central sensitization [87]. In addition to that, the contribution of altered calcium intracellular homeostasis due to mitochondrial dysfunction and for altered sodium/potassium conductance secondary to Na^+^-K^+^-ATPase dysfunction has also been confirmed [88,89]. Carfilzomib, a second-generation proteasome inhibitor approved for multiple myeloma treatment, substantially mirrors the bortezomib toxicity profile although it has been reported to have overall less neurotoxicity [77].

**Figure 6:**
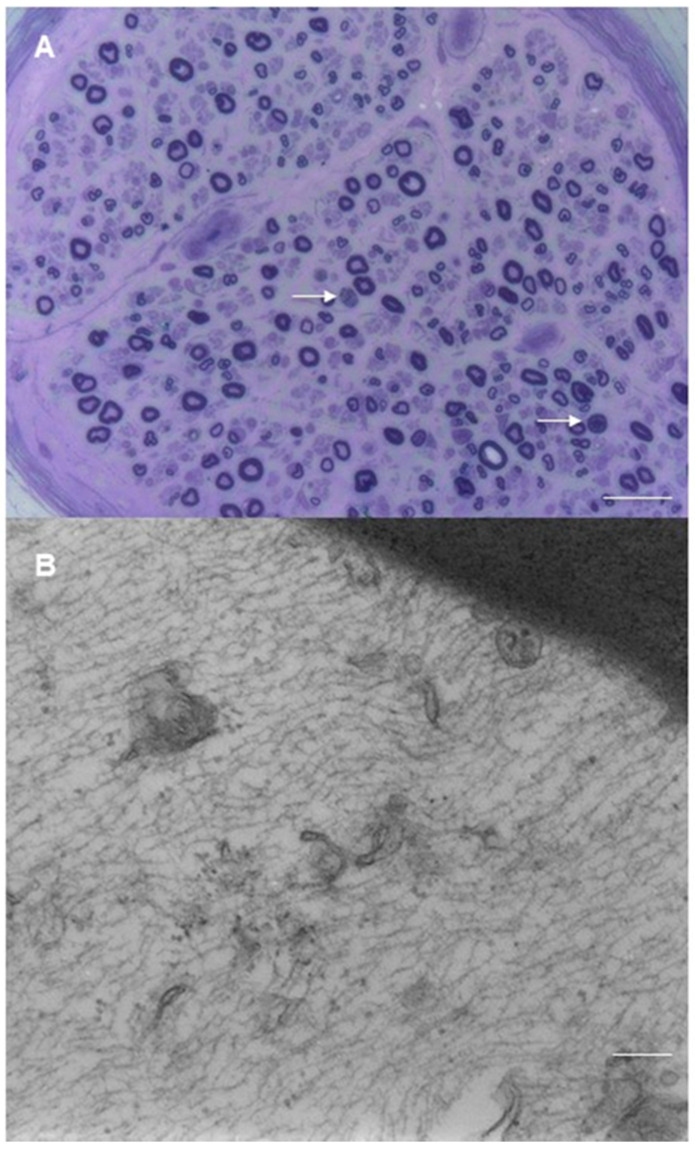
Sural nerve biopsy from a patient with brentuximab vedotin-related neuropathy. (**A**) axonal neuropathy is characterized by very mild loss of myelinated fibers; rare wallerian degenerations are present (arrow) (semithin section, toluidine blue, original magnification 40×; bar 50 mm). (**B**) A myelinated fiber shows an altered composition of cytoskeleton constitutes: is evident a relative preservation of neurofilaments and a significative depletion of microtubules (original magnification 30,000×; bar 300 nm).

Brentuximab vedotin is an anti-CD30 monoclonal antibody conjugated with monomethyl auristatin E (a tubulin polymerization inhibitor leading to cell apoptosis), included in therapeutic protocols for both B and T cell lymphomas. Besides the reports of an early sensory-motor axonal peripheral neuropathy, brentuximab may be associated with severe and rapidly progressing motor neuropathies, with histopathological studies disclosing diffuse depletion of axonal microtubules and the detection of misaligned neurofilaments [90,91,92] (Figure 6).

The immune checkpoint inhibitors (i.e., nivolumab and pembrolizumab), approved for relapsed/refractory Hodgkin’s lymphomas, act by targeting programmed cell death protein 1 (PD-1), a cell surface receptor with a crucial modulatory role on the immune system. Given their more recent introduction among the therapeutic options for hematological disorders, the incidence, clinical characteristics, and timing of possible iatrogenic peripheral neuropathy are still being defined [93,94]. However, a recent paper by Psimaras et al. showed that these drugs are considered relatively safe with regards to peripheral nerve involvement with a reported overall incidence of around 1.2–1.3% [95]. An immune-mediated demyelinating polyradiculoneuropathy has been reported in some patients undergoing treatment with immune checkpoint inhibitors but epidemiological data are still unreliable due to significant differences across studies. To date, few pathological studies have been performed on these patients, with evidence of epineurial perivascular inflammatory deposits (T lymphocytes) [96,97].

## 8. Conclusions

**Figure 7:**
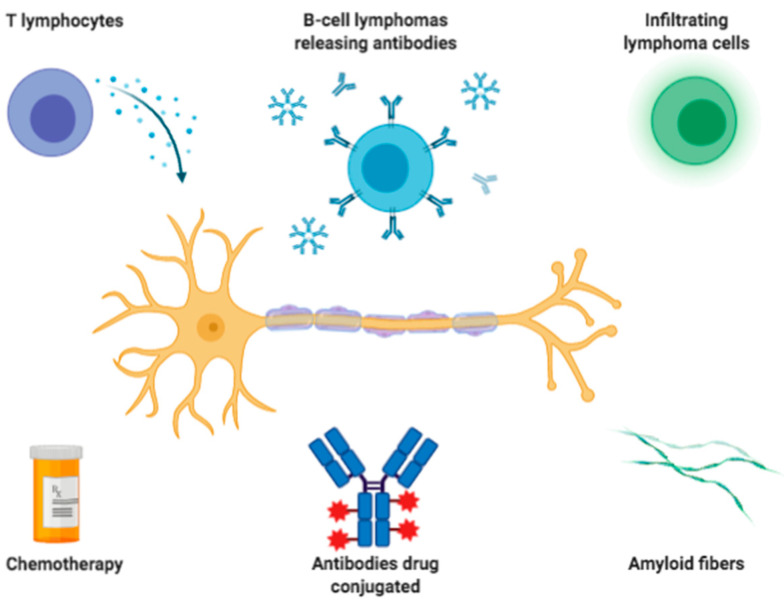
Main mechanisms of nerve damages of neuropathies associated with hematological diseases. Nerves, either myelin and/or axons, of patients with hematological diseases, might be damaged by cytotoxic T lymphocytes, antibodies released by neoplastic B-cell, infiltrating lymphoma cells, chemotherapy (i.e., vinca alkaloids, immunomodulating agents or protease inhibitors), antibodies drug-conjugated (like brentuximab vedotin) or the deposition of beta-amyloid fibers from free-light chains. Created online with BioRender.com.

Despite the availability of less invasive techniques, sural nerve biopsy constitutes an important diagnostic tool in the work-up of peripheral neuropathies, especially when associated with hematological conditions (Figure 7). In a few of them, namely neurolymphomatosis or vasculitis, nerve biopsies remain the gold standard. Moreover, neuropathological studies may help to clarify the pathogenic mechanisms in these disorders, possibly suggesting potential therapeutic approaches.

## Supplementary Materials

The following are available online at https://www.mdpi.com/2076-3425/11/2/132/s1, Table S1: Summary table of nerve biopsy findings in peripheral neuropathies associated with hematological conditions.
