# Supplementary material for: Mechanisms of Nerve Damage in Neuropathies Associated with Hematological Diseases: Lesson from Nerve Biopsies
Source: Brain Sci. 2021 Jan 20;11(2):132. doi: 10.3390/brainsci11020132 (PMC7909400; doi:10.3390/brainsci11020132)
Supplement: Supplementary file 1 [file brainsci-11-00132-s001.pdf]

Supplementary Files

Table S1. Summary table of nerve biopsy findings in peripheral neuropathies associated with hematological conditions.

	Histological findings on semithin section	Histological findings on paraffin section	Ultrastructural findings	Immunohistochemistry	Other findings
Anti-MAG	De-remyelination; Intramyelin oedema	-	Uncompaction myelin lamellae	Deposition of IgM and C3d fragment on myelin sheaths	TF: pale focal swellings
Cryoglobulinemia	Asymmetrical axonal neuropathy; petechiae	Epi-endoneurial pericapillaries inflammatory infiltrates; extravasation of red blood cells (petechiae)	Perivascular cryoglobulin deposits	Deposition of IgM or IgG or IgA according to cryoglobulinemia type	
Neurolymphomatosis	Axonal neuropathy, diffuse or focal; lympho-monocytes infiltrates	From rare to massive lymphocytic infiltrations, with abnormal monomorphic histopathological features	Detection of nuclear atypia	Immunophenotype characterization of neoplastic cells; detection of interspersed reactive T cells	Demonstration of monoclonality by PCR-Based clonality analysis
AL- Amyloidosis	Axonal neuropathy; pericapillary amorphous deposits	Congo Red staining: green apple birefringence at polarized light	Detection of nonbranching fibril with a diameter of 7.5–10 nm	Deposits of lambda or kappa chains	
POEMS	Axonal neuropathy with some de-remyelinated fibers; endoneurial oedema		Thickened basal lamina of hypertrophic and hyperplastic endothelial cells with narrowing of endoneurial vessels lumen		
CIPN	Neuronopathy or Axonal neuropathy		In some neuropathies, disarranged axonal cytoskeleton organization		

TF: teased fibers.
